# Combined Use of Waist and Hip Circumference to Identify Abdominally Obese HIV-Infected Patients at Increased Health Risk

**DOI:** 10.1371/journal.pone.0062538

**Published:** 2013-05-20

**Authors:** Trevor O’Neill, Giovanni Guaraldi, Gabriella Orlando, Federica Carli, Elisa Garlassi, Stefano Zona, Jean-Pierre Després, Robert Ross

**Affiliations:** 1 School of Kinesiology and Health Studies, Queen’s University, Kingston, Ontario, Canada; 2 Metabolic Clinic, Infectious and Tropical Disease Unit, Department of Medicine, University of Modena and Reggio Emilia, Modena, Italy; 3 Québec Heart Institute, Hôpital Laval Research Centre, Quebec City, Quebec, Canada; Rush University, United States of America

## Abstract

**Objectives:**

To determine whether for a given waist circumference (WC), a larger hip circumference (HC) was associated with a reduced risk of insulin resistance, type 2 diabetes (T2D), hypertension and cardiovascular disease (CVD) in HIV-infected patients. A second objective was to determine whether, for a given WC, the addition of HC improved upon estimates of abdominal adiposity, in particular visceral adipose tissue (VAT), compared to those obtained by WC alone.

**Methods:**

HIV-infected men (N = 1481) and women (N = 841) were recruited between 2005 and 2009. WC and HC were obtained using standard techniques and abdominal adiposity was measured using computed tomography.

**Results:**

After control for WC and covariates, HC was negatively associated with risk of insulin resistance (*p*<0.05) and T2D [Men: OR = 0.91 (95% CI: 0.86–0.96); Women: OR = 0.91 (95% CI: 0.84–0.98)]. For a given WC, HC was also negatively associated with a lower risk of hypertension (*p*<0.05) and CVD [OR = 0.94 (95% CI: 0.88–0.99)] in men, but not women. Although HC was negatively associated with VAT in men and women after control for WC (*p*<0.05), the addition of HC did not substantially improve upon the prediction of VAT compared to WC alone.

**Conclusions:**

The identification of HIV-infected individuals at increased health risk by WC alone is substantially improved by the addition of HC. Estimates of visceral adipose tissue by WC are not substantially improved by the addition of HC and thus variation in visceral adiposity may not be the conduit by which HC identifies increased health risk.

## Introduction

Waist (WC) and hip circumference (HC) have independent and opposite associations with type 2 diabetes (T2D) [1], [2], [3], [4], [5], [6], [7] and cardiovascular disease (CVD) [4], [6], [8], [9], [10], [11], [12], [13] in the general population. For a given WC, an increase in HC is associated with a reduction in health risk, whereas for a given HC a larger WC is associated with an increase in health risk. More importantly, it appears that the combination of WC and HC identify adults at increased risk for morbidity and mortality better than either alone [7], [9], [10], [13], [14], [15], [16]. Whether the combination of WC and HC can improve upon the identification of HIV-infected patients at increased health risk compared to WC alone is unknown.

Mechanisms that explain why HC is negatively associated with increased cardiometabolic risk for a given WC are unclear. One of the emerging mechanisms suggests that the increase in morbidity associated with a lower HC for a given WC may be explained by the presence of dysfunctional, insulin-resistant peripheral subcutaneous adipocytes [17]. This could lead to impaired storage of excess energy and thus a reduction in adipose tissue mass reflected by a smaller HC or thigh circumference (ThC). Accordingly lower-body subcutaneous adipose tissue may act as a buffer by storing excess energy thus avoiding lipid spillover into ectopic tissues, such as visceral adipose tissue (VAT) [17]. Indeed, we have previously reported that HC is negatively associated with VAT after control for WC in non-HIV individuals [18]. Confirmation in HIV-infected patients treated with antiretroviral therapy (ART), a known component of adipocyte dysfunction [19], would provide at least one mechanism by which a reduction in HC for a given WC is associated with an increase in health risk.

Based on these observations we reasoned that the combination of WC and HC or ThC may provide an improved, pragmatic approach for identifying HIV-infected patients at increased health risk. Given the established association between VAT and morbidity in HIV-infected individuals, we also determined whether for a given WC, the addition of HC or ThC improved upon estimates of VAT compared to those obtained by WC alone.

## Methods

### Subjects

Participants included 2322 HIV-infected patients (1481 men and 841 women) recruited at the metabolic clinic of the University of Modena and Reggio Emilia in Italy between 2005 and 2009. Patients from HIV clinics throughout Italy are referred or have direct access to the multidisciplinary treatment at the metabolic clinic where they obtain comprehensive metabolic and anthropometric diagnostic and therapeutic assessments for the presence of lipodystrophy and non-infectious comorbidities.

Inclusion criteria were serologically documented HIV-1 infection, age >18 years and at least 18 months of ART exposure. A signed informed-consent form to participate in this study was obtained from each patient. The study was approved by the local institutional review board (Comitato Etico Provinciale di Modena).

### Demographics

Demographic and clinical data, including duration of HIV infection, ART history, and lifestyle were obtained by medical chart review. Smoking was separated as follows: non-smoker (<1 cigarettes per day) or smoker (≥1 cigarettes per day). Alcohol consumption was separated into no alcohol (<10 g of ethanol per day) or alcohol (≥10 g of ethanol per day). Physical activity was defined as mild or intense when <4 or ≥4 hours per week of exercise, respectively, were reported.

### Anthropometric Measurements

All patients underwent physical examinations on the day fasting blood was obtained. WC was measured at the narrowest point mid-way between the lowest rib and the iliac crest at the end of expiration with the subject standing. HC was taken at the largest point at the level of the greater trochanters, and thigh circumference was measured mid-way between the hip and knee. All circumference measures were calculated as the average of three measurements. BMI was calculated as weight in kilograms divided by the square of height in meters.

### Body Composition

A single CT image at the level of the L4 vertebra was taken for quantification of VAT and abdominal subcutaneous adipose tissue using a 64-multislice CT scanner (LightSpeed VTC; General Electric Medical System). Each CT image was analyzed using software application based on Advantage Windows 4.4 GE medical system. Two radiologists assessed CT images for VAT and abdominal subcutaneous adipose tissue. Agreement between operators for VAT and abdominal subcutaneous adipose tissue measurements was calculated on a subset of 40 scans and demonstrated a high repeatability (r = 0.97, β = 0.98 and r = 0.97 and β = 1.01 respectively). The mean difference for VAT and abdominal subcutaneous adipose tissue was −0.5±0.8 and 0.4±0.8 cm^3^ respectively.

### Cardiometabolic Risk Factors

Total cholesterol, low-density lipoprotein cholesterol, high-density lipoprotein cholesterol, triglyceride, glucose, and insulin levels were measured after an overnight fast. Insulin resistance was calculated using the homeostasis model assessment equation (HOMA-IR = [fasting insulin (mU/ml) × fasting glucose (mmol/L)/22.5].

### Criteria for Disease

A cut-point of HOMA-IR ≥3 was used to define insulin resistance. T2D was diagnosed according to the American Diabetes Association criteria, past medical history of diabetes or use of glucose lowering therapy [20]. Hypertension was defined as a systolic pressure ≥140 mmHg or a diastolic pressure ≥90 mmHg. Cardiovascular events (CVD) were defined as myocardial infarction, revascularization, stroke, and/or peripheral vascular disease that occurring either before or after the clinical evaluation within a 5-year period. This was chosen to increase the statistical power of the sample size calculation.

### Statistical Analyses

All statistical analyses were performed separately for men and women due to gender interactions as well as the well-known differences in body shape between men and women. Patient characteristics were compared between men and women: categorical variables were analyzed using Chi-squared test, while continuous variables were compared using Student’s T-test or Mann-Whitney test where appropriate. Events were coded as 0 (no event) and 1 (event). Univariate and multivariate logistic regression analyses were used to determine the associations between WC, HC, ThC and BMI with HOMA-IR, T2D, hypertension and CVD. Odds ratios (OR) with 95% confidence intervals (CI) are expressed after adjustment for covariates. For all events, age, smoking, and physical activity were included as covariates in the analyses. Univariate and multivariate linear regressions were performed to determine the independent associations between WC, HC, ThC and BMI with total abdominal adipose tissue, VAT and abdominal subcutaneous adipose tissue. All statistical analyses were performed using STATA 12.1 Intercooled version for Mac, StataCorp, College Station, TX, USA.

## Results

Despite similarity in age (Men 45.9±7.3, Women 43.1±6.9) and presenting with generally lower circumference values, the women were characterized by greater subcutaneous adipose tissue and less lean mass compared to men (Table 1). As expected men had more VAT while women had more abdominal subcutaneous adipose tissue. In general women presented with more favorable lipid profiles compared to men. The prevalence rates were 44.8% and 37.9% for HOMA-IR, 9.1% and 5.7% for T2D, 43.1% and 23.6% for hypertension and 5.1% and 1.6% for CVD in men and women, respectively (Table 1). Sixty-two CVD events were reported before and 27 events within two years after the clinical evaluation. Women had a marginally longer exposure to ART compared to men, while levels of CD4 did not differ between the two groups.

**Table 1:** Patient characteristics.

	Men (*n* = 1481)	Women* (*n = *841)
**Demographics**		
Age, y	45.9±7.3	43.1±6.9
Smoke (≥1 cigs/day), %	47.1	49.0
Alcohol Consumption, %	49.3	39.8
Physical Activity, %	36.1	27.8
**Anthropometrics**		
BMI, kg/m^2^	24.1±3.8	22.5±4.0
Waist circumference, cm	88.0±10.1	82.7±10.3
Thigh circumference cm	47.8±4.3	44.8±4.9
Hip circumference, cm	90.0±6.3	88.8±7.3
Waist-to-hip ratio	0.98±0.07	0.93±0.7
**Body Composition**		
Computed Tomography (cm^3^)		
VAT	143.9±88.8	105.0±91.9
Abdominal subcutaneousadipose tissue	121.4±96.3	183.1±11.1
DEXA (kg)		
Total fat	10.6±6.4	13.6±6.9
Arm fat	1.7±1.3	2.4±1.6
Arm lean	7.6±1.4	4.8±1.2
Leg fat	2.2±1.8	3.3±2.4
Leg lean	17.9±2.7	12.7±1.9
**Cardiovascular Risk Factors**		
Total Cholesterol, mmol/L	4.8±1.2	4.9±1.3
Triglycerides, mmol/L	2.5±2.0	1.8±1.2
HDL, mmol/L	1.1±0.4	1.3±0.4
LDL, mmol/L	2.9±1.0	3.0±1.0
HOMA-IR	4.6±5.8	3.9±4.0
Fasting Glucose, mmol/L	5.4±1.2	5.1±1.1
Fasting Insulin, mU/mL	18.3±18.6	16.5±14.3
**Outcomes**		
HOMA-IR≥3, %	44.8	37.9
Type 2 diabetes, %	9.2	5.7
Hypertension, %	43.1	23.6
Cardiovascular disease, %	5.1	1.6
**HIV**		
Exposure to ART, months	101 (51–134)	108 (65–141)
Current Thimidine analogue treatment, %	7.5	10.9
CD4	556.9±267.4	552.3±259.2

Data presented as percent, mean ± SD, or median (interquartile range).

* With the exception of LDL and CD4, differences between sexes were significant (*p*≤0.05).

### Anthropometric Associations with HOMA-IR, T2D, Hypertension and CVD

Univariate analyses revealed that WC was positively associated with HOMA-IR, hypertension and CVD in men and women as well as T2D in men (Table 2). HC was positively associated with HOMA-IR and hypertension in men and women. With the exception of HOMA-IR, thigh circumference alone was not associated with any cardiometabolic risk factor or morbidity.

**Table 2:** Univariate associations between WC, HC, thigh circumference, BMI and WHR with HOMA-IR, T2D, hypertension and CVD.

	Men		Women	
	OR	95% CI	OR	95% CI
**HOMA-IR≥3**				
WC	1.06*	1.05–1.08	1.06*	1.04–1.08
HC	1.06*	1.03–1.08	1.04*	1.01–1.06
ThC	1.04*	1.02–1.07	1.03*	1.00–1.07
BMI	1.16*	1.12–1.21	1.17*	1.12–1.23
WHR	1.06*	1.05–1.08	1.04*	1.03–1.07
**T2D**				
WC	1.03*	1.02–1.05	1.02	0.99–1.05
HC	1.02	0.98–1.05	0.99	0.95–1.04
ThC	0.99	0.95–1.04	0.92	0.88–1.01
BMI	1.07*	1.02–1.12	1.06	0.99–1.13
WHR	1.08*	1.05–1.11	1.05*	1.01–1.09
**Hypertension**				
WC	1.04*	1.03–1.05	1.04*	1.02–1.06
HC	1.04*	1.02–1.06	1.04*	1.01–1.06
ThC	1.01	0.99–1.04	1.03	0.99–1.06
BMI	1.08*	1.05–1.12	1.11*	1.07–1.15
WHR	1.06*	1.05–1.08	1.04*	1.02–1.07
**CVD**				
WC	1.04*	1.02–1.06	1.05*	1.01–1.10
HC	1.03	1.00–1.07	1.06*	1.01–1.12
ThC	1.00	0.95–1.05	1.00	0.89–1.12
BMI	1.07*	1.02–1.13	1.11*	1.01–1.22
WHR	1.08*	1.05–1.11	1.04	0.96–1.11

Abbreviations: BMI, body mass index; CVD, cardiovascular disease; HC, hip circumference; ThC, thigh circumference; WC, waist circumference; T2D, type 2 diabetes; WHR, waist-to-hip-ratio (per 0.01 increase).

* *p*≤0.05.

Multivariate associations between WC, HC, ThC and BMI with HOMA-IR, T2D, hypertension and CVD are shown in Table 3. Whereas for a given WC, HC attenuated the risk for cardiometabolic risk factors and morbidity, for a given HC, WC increased the risk. Indeed, for a given WC, a 1 cm greater HC was associated with a 7–9% reduction in risk for HOMA-IR and T2D in men and women. Whereas for a given HC, a 1 cm greater WC was associated with a 5–13% increase in risk for HOMA-IR and T2D in men and women compared to a 3–8% increase in risk when WC was used alone. In men, WC and HC also showed independent and inverse associations with hypertension and CVD (Table 3). For a given WC, a 1 cm larger HC was associated with a 4–6% reduction in risk for hypertension and CVD in men. Whereas, for a given HC, a 1 cm larger WC was associated with a 5–6% increase in risk for hypertension, and CVD in men compared to a 4% increase in risk when WC was used alone.

**Table 3:** Multivariate associations between WC, HC, thigh circumference and BMI with HOMA-IR, T2D, hypertension and CVD.

	Men		Women	
	WC	HC	WC	HC
**HOMA-IR**				
Model 1	1.14* (1.11–1.17)	0.90* (0.86–0.94)	1.11* (1.08–1.15)	0.93* (0.89–0.96)
Model 2	1.13* (1.10–1.16)	0.91* (0.87–0.95)	1.11* (1.08–1.14)	0.93* (0.89–0.97)
**T2D**				
Model 1	1.09* (1.05–1.13)	0.90* (0.85–0.96)	1.07* (1.02–1.12)	0.92* (0.85–0.98)
Model 2	1.07* (1.03–1.11)	0.91* (0.86–0.96)	1.05* (1.01–1.10)	0.91* (0.84–0.98)
**Hypertension**				
Model 1	1.06* (1.04–1.09)	0.96* (0.93–0.99)	1.05* (1.02–1.07)	0.99 (0.95–1.02)
Model 2	1.05* (1.03–1.07)	0.96* (0.93–0.99)	1.04* (1.01–1.07)	0.98 (0.95–1.02)
**CVD**				
Model 1	1.08* (1.04–1.12)	0.93* (0.87–0.99)	1.03 (0.96–1.11)	1.03 (0.94–1.13)
Model 2	1.06* (1.02–1.11)	0.94* (0.88–0.99)	1.03 (0.95–1.11)	1.02 (0.92–1.14)
	**WC**	**ThC**	**WC**	**ThC**
**HOMA-IR**				
Model 3	1.10* (1.08–1.12)	0.93* (0.89–0.97)	1.08* (1.06–1.11)	0.94* (0.90–0.98)
Model 4	1.08* (1.06–1.11)	0.97 (0.93–1.01)	1.08* (1.06–1.11)	0.94* (0.90–0.98)
**T2D**				
Model 3	1.05* (1.03–1.08)	0.92* (0.87–0.98)	1.05* (1,02–1.08)	0.88* (0.83–0.95)
Model 4	1.03* (1.00–1.05)	0.96 (0.90–1.02)	1.03 (0.99–1.06)	0.91* (0.85–0.98)
**Hypertension**				
Model 3	1.05* (1.04–1.07)	0.95* (0.92–0.97)	1.04* (1.02–1.06)	0.98 (0.95–1.02)
Model 4	1.04* (1.02–1.05)	0.97* (0.93–1.00)	1.03* (1.01–1.05)	1.01 (0.97–1.05)
**CVD**				
Model 3	1.06* (1.04–1.09)	0.92* (0.86–0.98)	1.07* (1.02–1.12)	0.94 (0.84–1.05)
Model 4	1.04* (1.01–1.07)	0.96 (0.90–1.03)	1.05 (0.99–1.10)	0.97 (0.86–1.10)
	**WC**	**BMI**	**WC**	**BMI**
**HOMA-IR**				
Model 5	1.07* (1.03–1.10)	1.03 (0.95–1.12)	1.04* (1.01–1.08)	1.07 (0.97–1.17)
Model 6	1.04* (1.00–1.07)	1.12* (1.02–1.22)	1.05* (1.01–1.08)	1.07 (0.97–1.17)
**T2D**				
Model 5	1.05* (1.01–1.10)	0.95 (0.85–1.06)	1.00 (0.95–1.06)	1.05 (0.93–1.20)
Model 6	1.03 (0.98–1.07)	0.98 (0.87–1.10)	0.99 (0.93–1.05)	1.05 (0.92–1.22)
**Hypertension**				
Model 5	1.06* (1.04–1.09)	0.94* (0.89–0.99)	1.01 (0.98–1.04)	1.07* (1.00–1.16)
Model 6	1.04* (1.02–1.07)	0.96 (0.90–1.02)	1.01 (0.98–1.04)	1.08* (1.00–1.16)
**CVD**				
Model 5	1.08* (1.03–1.13)	0.90 (0.79–1.02)	1.05 (0.96–1.15)	0.99 (0.80–1.24)
Model 6	1.05 (1.00–1.10)	0.94 (0.83–1.08)	1.04 (0.94–1.14)	1.01 (0.80–1.27)

Model 1 includes WC and HC as independent variables.

Model 2 includes WC, HC and covariates (age, physical activity, smoking) as independent variables.

Model 3 includes WC and thigh circumference (ThC) as independent variables.

Model 4 includes WC, ThC and covariates (age, physical activity, smoking) as independent variables.

Model 5 includes WC and BMI as independent variables.

Model 6 includes WC, BMI and covariates (age, physical activity, smoking) as independent variables.

Data presented as OR (95% CI).

Abbreviations: BMI, body mass index; CVD, cardiovascular disease; HC, hip circumference; ThC, thigh circumference; WC, waist circumference; T2D, type 2 diabetes.

* *p*≤0.05.

With few exceptions, the addition of ThC or BMI to WC was not associated with a reduction in risk for HOMA-IR, T2D, hypertension or CVD in men and women (Table 3).

### Anthropometric Associations with VAT

WC, HC, ThC and BMI were positively associated with total abdominal adipose tissue, VAT and abdominal subcutaneous adipose tissue in both men and women (Table 4). Following adjustment for WC, the associations between HC and ThC with VAT were inversed, such that HC and thigh circumference were negatively associated with VAT in men and women (Table 5). HC in men and women as well as ThC in men remained significant correlates of VAT after further control for age. Although for a given WC HIV-infected patients with a lower HC or ThC had increased levels of VAT, the increase was not substantial by comparison to WC alone. For example, in men, the inclusion of HC or ThC added just 3% to the variance explained for VAT, while in women, the inclusion of HC and ThC increased the variance explained for VAT by 1–2%. In general, similar observations were observed for total abdominal adipose tissue and abdominal subcutaneous adipose tissue (Table 5).

**Table 4:** Univariate associations between WC, HC, thigh circumference, BMI and WHR with total abdominal adipose tissue, VAT and abdominal subcutaneous adipose tissue.

	Men		Women	
	β	r^2^	β	r^2^
**TAAT**				
WC	12.73	0.70	11.59	0.50
HC	17.54	0.51	14.05	0.37
ThC	16.61	0.22	14.16	0.17
BMI	31.93	0.60	28.20	0.44
WHR	14.96	0.41	10.50	0.21
**VAT**				
WC	5.44	0.38	3.97	0.20
HC	6.54	0.23	3.37	0.07
ThC	5.64	0.08	2.95	0.03
BMI	12.84	0.30	8.23	0.12
WHR	7.33	0.30	4.97	0.16
**ASAT**				
WC	7.30	0.59	7.62	0.50
HC	11.00	0.52	10.68	0.50
ThC	11.00	0.24	11.21	0.24
BMI	19.08	0.56	19.97	0.52
WHR	7.63	0.28	5.53	0.14

Abbreviations: ASAT, abdominal subcutaneous adipose tissue; BMI, body mass index; HC, hip circumference; ThC, thigh circumference; TAAT, total abdominal adipose tissue; VAT, visceral adipose tissue; WC, waist circumference; WHR, waist-to-hip ratio.

All associations were significant (*p*<0.001).

**Table 5:** Multivariate associations between WC, HC, thigh circumference and BMI with total abdominal adipose tissue, VAT and abdominal subcutaneous adipose tissue.

	Men		Women	
	β	r^2^	β	r^2^
**TAAT**				
*Model 1*				
WC	11.67*	0.70	9.40*	0.51
HC	2.04*		4.02*	
*Model 2*				
WC	12.85*	0.69	10.73*	0.51
ThC	−0.43		3.65*	
*Model 3*				
WC	10.12*	0.70	8.10*	0.52
BMI	7.88*		10.57*	
*Model 4*				
WHR	14.8*	0.41	10.3*	0.21
**VAT**				
*Model 1*				
WC	6.00*	0.41	4.90*	0.22
HC	−1.70*		−1.99*	
*Model 2*				
WC	5.44*	0.41	4.02*	0.21
ThC	−1.35*		−0.82	
*Model 3*				
WC	4.88*	0.41	4.21*	0.21
BMI	0.73		−1.17	
*Model 4*				
WHR	6.78*	0.32	4.80*	0.18
**ASAT**				
*Model 1*				
WC	5.67*	0.63	4.49*	0.57
HC	3.75*		6.01*	
*Model 2*				
WC	7.41*	0.61	6.71*	0.53
ThC	0.92*		4.48*	
*Model 3*				
WC	5.24*	0.63	3.89*	0.56
BMI	7.16*		11.74*	
*Model 4*				
WHR	8.01*	0.29	5.56*	0.14

Model 1 includes WC, HC and age as independent variables.

Model 2 includes WC, thigh circumference and age as independent variables.

Model 3 includes WC, BMI and age as independent variables.

Model 4 includes WHR and age as independent variables.

Abbreviations: ASAT, abdominal subcutaneous adipose tissue; BMI, body mass index; HC, hip circumference; ThC, thigh circumference; TAAT, total abdominal adipose tissue; VAT, visceral adipose tissue; WC, waist circumference, WHR, waist-to-hip ratio.

* *p*≤0.05.

## Discussion

The primary finding of this study was that the identification of HIV-infected patients at increased risk by WC alone is substantially improved by the addition of HC. These observations reinforce the recommendation that WC and HC should be used in combination to identify higher-risk HIV-infected adults, in particular men, and that identification of HIV-infected adults with elevated health risk may be underestimated when WC is used alone. Our observations also suggest that the ability of WC and HC combined to predict VAT might not be the conduit by which these anthropometric measures combined identify those at increased risk of cardiometabolic risk and morbidity.

To our knowledge no prior study has determined whether HC adds to the health risk identified by WC alone in HIV-infected patients. Our finding is consistent with prior studies in non-HIV individuals investigating the associations of WC and HC with morbidity, which have also demonstrated a protective effect of an increased HC for a given WC and/or BMI [2], [4], [7], [13], [21]. For example, Sakai et al. [21] reported that upon control for WC in men and women, the odds ratios for hypertension and T2D ranged from 0.88 to 0.94 per 1 cm increase in HC, which are of similar magnitude to those reported here.

The importance of our primary finding can be appreciated by comparing the health risk of a 1 cm difference in both WC and HC with a 1 cm difference in WC alone. If an HIV-infected patient has a 1 cm greater WC and a 1 cm lower HC compared to another HIV-infected patient, the risk of HOMA-IR and T2D using WC and HC will increase 14–22% in men and women, while the risk is just 3–8% using WC alone. Similarly in an HIV-infected man, a 1 cm greater WC combined with a 1 cm lower HC, compared to another HIV-infected man, will increase the risk of hypertension and CVD 9–12% using WC and HC whereas the use of WC alone is associated with an increased risk approximating 4%. Given that lower body subcutaneous adipose tissue is a common site for loss of adipose tissue in HIV-infected adults [22], [23], [24], a strong argument exists for the routine acquisition of WC and HC in clinical settings.

Our findings that derive from the use of simple anthropometric tools that are readily available to the practitioner are consistent with studies in HIV-infected patients wherein sophisticated radiographic methods were used [25], [26], [27], [28]. For example, Mynarcik et al. [26] previously showed that after control for trunk adipose tissue, the loss of peripheral adipose tissue measured by DXA was strongly associated with insulin resistance. Similarly, Lake et al. [28] reported that leg subcutaneous adipose tissue measured by magnetic resonance imaging was negatively associated with CVD risk following control for VAT.

The mechanism that would explain why HC is negatively associated with increased cardiometabolic risk and morbidity following control for WC is unclear. An emerging hypothesis is that dysfunctional, insulin-resistant subcutaneous adipocytes are unable to entrap extra energy which may result in a spillover into ectopic tissues, such as the liver, the heart, the skeletal muscle or VAT [17]. Therefore, lower-body subcutaneous adipose tissue may play a protective role by acting as a buffer through storage of excess lipids thus limiting lipid deposition in ectopic tissues. Treatment with ART has been shown to cause mitochondrial dysfunction and enhanced lipolysis as well as impaired glucose uptake in subcutaneous adipose tissue, which may lead to adipocyte dysfunction or apoptosis [19], [29]. Thus lower HC values in HIV-infected patients may indicate the presence of dysfunctional lower-body adipocytes which are unable to store excess energy resulting in a decrease in adipose tissue mass. Presumably this would lead to a greater accumulation of lipid in ectopic tissues including VAT.

Consistent with this hypothesis we observed that HC was negatively associated with VAT after control for WC, a finding consistent with previous observations in non-HIV adults [18]. However, the magnitude of the increase in VAT predicted by the combination of WC and HC compared to WC alone was subtle averaging 3% (∼4 cm^3^) in men and 2% (∼2 cm^3^) in women. It is unlikely that differences in VAT estimation of this magnitude would mediate the incremental increase in health risk identified by HC. It is possible that HC in combination with WC identifies ectopic fat deposition in tissues other than VAT including liver and/or epicardial tissues both of which are associated with increased health risk [30]. Although the mechanisms that explain why health risk is substantially elevated by the combination of WC and HC compared to WC alone remain to be determined, it is noted that numerous studies report an inverse association between lower-body adipose tissue and morbidity after control for abdominal adiposity in non-HIV adults [2], [6], [7], [10], [13], [21].

Although ThC was negatively associated with all our outcomes after control for WC independent of gender, unlike HC the associations did not persist after control for covariates. This was a surprising result because we had no reason to believe that the anthropometric variables represent adipose tissue depots that vary metabolically. A threshold value for ThC that delineates increased morbidity risk in HIV-infected persons is unknown, however, the ThC values for our sample (∼46 cm) is well below the ThC threshold for CVD (∼60 cm) reported for non-HIV men and women [12]. The recent observation that lipoatrophy as measured by leg SAT is negatively associated with CVD risk independent of VAT in HIV-infected adults [28] suggests that further research is warranted to determine the optimal use of ThC in clinical practice.

Our study is cross-sectional in design and causality cannot be assumed. Further, whether simultaneous changes in WC and/or HC over time identify increased risk of morbidity cannot be inferred. The subjects studied were referred to the HIV clinic with specific metabolic disorders and thus generalizability of our findings across the HIV population is unclear. The relatively small sample size precludes the derivation of algorithms and/or cut-point values for HC in combination with WC that would be required to identify those at increased health risk.

Our findings demonstrate that the identification of HIV-infected individuals at increased health risk by WC alone is substantially improved by the addition of HC. Further study with large sample sizes is required to develop valid algorithms or cut-point values that could ultimately be incorporated into routine clinical practice to help identify high-risk HIV patients.
